# Maternal perception of factors that interfere with breastfeeding of preterm newborns

**DOI:** 10.1590/2317-1782/20242023252en

**Published:** 2024-08-05

**Authors:** Bárbara Helem da Fonseca Patrocínio Werneck, Juliana Cordeiro de Oliveira, Cláudia Gonçalves de Oliveira, Andréa Rodrigues Motta, Amélia Augusta de Lima Friche, Renata Maria Moreira Moraes Furlan

**Affiliations:** 1 Graduação em Fonoaudiologia, Universidade Federal de Minas Gerais – UFMG - Belo Horizonte (MG), Brasil.; 2 Programa de Pós-graduação em Ciências Fonoaudiológicas, Universidade Federal de Minas Gerais - UFMG - Belo Horizonte (MG), Brasil.; 3 Departamento de Fonoaudiologia, Universidade Federal de Minas Gerais – UFMG - Belo Horizonte (MG), Brasil.

**Keywords:** Breast Feeding, Infant, Premature, Neonatology, Speech, Language and Hearing Sciences, Maternal and Child Health

## Abstract

**Purpose:**

to verify the association between the perception of mothers of premature infants regarding the features that may interfere with breastfeeding and the mother's socioeconomic data, pregnancy and the baby's clinical data.

**Methods:**

observational, descriptive and analytical quali-quantitative cross-sectional study. One hundred and fourteen mothers of premature infants were included and data were collected through questionnaires, applied at hospital discharge, and analysis of medical records. Maternal responses about the interference observed in the breastfeeding process were categorized by content analysis and associated with socioeconomic, pregnancy and baby data.

**Results:**

the mothers' perceptions regarding the factors that interfere with the baby's feeding at the mother's breast were divided into four semantic categories: clinical and/or physical conditions of the baby; clinical, physical and/or psycho-emotional conditions of the mother; support network; and strategies for initiating and/or maintaining breastfeeding. Education, paternal presence, having other children and having breastfed them were associated with the maternal perception that their clinical, physical and/or psycho-emotional conditions interfere with breastfeeding. In addition, the support network was associated with exclusive breastfeeding at discharge.

**Conclusion:**

education, paternal presence, multiparity and having breastfed previous children influenced the maternal perception that their clinical, physical and/or psycho-emotional conditions interfere with breastfeeding. In addition, mention of the support network was associated with exclusive breastfeeding at discharge.

## INTRODUCTION

Prematurity is a risk factor for infant morbidity and mortality^(1)^. Premature newborns are those born before completing 37 weeks of gestation^(2)^. It can be classified, according to gestational age, as moderately-to-late preterm (32 weeks to 36 weeks and 6 days of gestational age), very preterm (28 weeks to 31 weeks and 6 days of gestational age), and extremely preterm (fewer than 28 weeks of gestational age)^(2)^. Very low gestational age, asphyxia, low birth weight, and sepsis are the highest risk factors for a poor prognosis and infant mortality^(3)^.

Preterm birth interrupts intrauterine development; thus, many of these newborns require care from different professionals to adapt to the external environment^(4)^. Nutrition is one of the challenges to preterm care in neonatal units, as these newborns may have certain nutritional and physiological limitations in the digestive system^(1)^.

Breastfeeding reduces infant morbidity and mortality, ensures a greater bond between mother and child^(5)^, and contributes to the adequate development of the stomatognathic system and its functions, such as sucking, breathing, mastication, swallowing, and speech articulation^(6)^. Feeding the child at the mother's breast promotes harmonious craniofacial growth and intra and extraoral muscle balance^(6)^.

Breastfeeding may start later and end sooner in preterm than in full-term newborns^(7)^. Monitoring mothers is important for successful breastfeeding, especially in the first days of the newborn's life and hospitalization, but also after hospital discharge^(8)^. Some factors can interfere with breastfeeding, regardless of prematurity, such as age, socioeconomic situation, the mother’s working and psychoemotional conditions, the newborn’s clinical conditions, hospital routines, and the mother’s support network, including the father, family, friends, and healthcare professionals^(9)^.

A study found that exclusive breastfeeding of preterm newborns at hospital discharge from two baby-friendly hospitals in Southeastern Brazil was influenced by marital status, maternal occupation, number of prenatal consultations, type of delivery, gestational age, birth weight, length of hospital stay, and use of mechanical ventilation^(10)^. In a public hospital in Maceió, maternal age over 35 years was a protective factor for exclusive breastfeeding in preterm newborns, while cesarean delivery was a risk factor^(11)^. In a maternity hospital in Paraná, extremely preterm birth and the use of pacifiers were predictive factors for early weaning (before the sixth month)^(12)^. Babies with a higher degree of prematurity were less likely to be exclusively breastfed at hospital discharge, as were those with lower birth weight, longer hospital stays, and who used mechanical ventilation^(13,14)^.

Hence, the opinion of preterm newborns’ mothers is essential to identify which of these existing factors favor or hinder breastfeeding. Such understanding helps seek strategies to directly address their needs and increase the chances of establishing and maintaining breastfeeding for these children*.*

Thus, this study aimed to verify the association of the mothers’ perception of factors that can interfere with preterm babies’ breastfeeding with the mothers’ socioeconomic and pregnancy characteristics and the newborns’ clinical status.

## METHOD

This descriptive, analytical, observational, qualitative-quantitative cross-sectional study was conducted in the neonatal unit of a public hospital in Betim, Minas Gerais, Brazil. The study sample comprised 114 mothers of preterm newborns, with no age limit for inclusion.

The study was approved by the Research Ethics Committee of the Federal University of Minas Gerais, under opinion number 3.589.241, and by the Research Ethics Committee of the City of Betim, Minas Gerais, under opinion number 4.222.766. All study participants signed an informed consent or assent form.

Data were collected between October 2019 and March 2020, considering the following inclusion criteria: the child being born in the maternity ward of the Professor Osaldo Franco Regional Public Hospital, being preterm (born at less than 37 weeks of gestational age), having been hospitalized for at least 48 hours in that hospital’s neonatal intensive care unit (NICU), and the mother desiring to breastfeed. The exclusion criteria were the child having been transferred to another institution, having a suspected or confirmed diagnosis of syndromes, periventricular or intraventricular hemorrhages grades III and IV, craniofacial malformation, malformation of the organs of the digestive system (e.g., cleft lip and palate and esophageal atresia), or abnormal breast milk absorption and digestion (e.g., infant galactosemia); and the mother having HIV/AIDS or cognitive incapacity to answer the questionnaire. This information was obtained from hospital records.

The study collected information on the mothers’ socioeconomic characteristics, such as age, education (no study, incomplete middle school, complete middle school, incomplete high school, complete high school, incomplete higher education, or complete higher education), whether they had a paid job, and whether the fathers were present in their daily lives. It also collected data on pregnancy (the number of previous pregnancies and whether the other children had been breastfed) and, close to the hospital discharge, the mothers' opinions on factors they believed interfere with the newborn's breastfeeding by either favoring or hindering it. The newborns’ data regarding weight, gestational age at birth, days of hospital stay, and diet at discharge were collected from the medical records. Their gestational age at birth served as a parameter to classify the degree of prematurity, according to the subcategories of the World Health Organization^(2)^.

These data were organized and stored in an Excel^®^ spreadsheet. Bardin’s category content analysis method^(15)^ was used to analyze the mothers’ responses regarding the factors that interfere with the newborn's feeding, as they are open questions. Category analysis consists of breaking down the text into smaller units – i.e., words, phrases, or expressions^(15)^.

Content analysis has three phases: pre-analysis; material exploration, categorization, or coding; and treatment of results, inferences, and interpretation^(15)^. Data were pre-analyzed by transcribing them into a spreadsheet and reading them in full. The categories were defined based on words or expressions mentioned repetitively by mothers. After identifying the most discussed topics, the researchers analyzed the results and defined the four semantic categories that group the mothers’ opinions. Each answer could be classified into more than one category, depending on the different factors that mothers considered to interfere with breastfeeding.

The descriptive statistical analysis was performed using tables with absolute and relative frequency distribution of categorical variables and numerical synthesis of quantitative variables. The dependent variables were the mothers' responses regarding their perceptions about the factors that interfere with breastfeeding. The independent variables were the mothers’ age, education, professional activity, primiparity, and previous breastfeeding experience; paternal presence; and newborns’ weight classification, preterm classification, days of hospital stay, and type of diet at discharge.

The Shapiro-Wilk test was initially used for the type of data distribution, verifying the absence of normality characteristics. The Pearson chi-square test verified the association between the categorical independent variables and the response variables related to the mother's responses. The Kruskal-Wallis test investigated the relationship between the mothers’ ages and responses. The significance level was set at 5% in all analyses. Education was categorized into up to incomplete high school (including incomplete and complete middle school and incomplete high school) and complete high school or higher (including complete high school and incomplete or complete higher education).

## RESULTS

The study included 114 mothers, aged 15 to 44 years, with a mean of 28.1 years and standard deviation (SD) of 7.8 years. They had a mean of 1.6 previous pregnancies (SD = 1.7); the minimum was 0, and the maximum was 9.

Table 1 presents the frequency analysis of the study's independent variables. Most mothers had completed high school, reported not working outside the home, reported that the child's father was present, had other children, and confirmed having breastfed their previous children. Most newborns were late preterm, had low weight, and had been hospitalized for up to 30 days, and just over half were discharged on exclusive breastfeeding.

**Table 1:** Descriptive analysis of the characteristics of the mother and their preterm newborns

Categorical variable	Absolute frequency	Relative frequency (%)
Education level		
Incomplete middle school	12	10.5
Complete middle school	10	8.8
Incomplete high school	33	28.9
Complete high school	57	50.0
Complete or incomplete higher education	2	1.7
Works outside the home		
Yes	46	40.3
No	68	59.6
The father is present		
Yes	103	90.3
No	11	9.6
Primiparity		
Primiparous	35	30.7
Multiparous	79	69.3
Breastfed previous children		
Yes	70	61.4
No	7	6.1
Not applicable	37	32.5
Birth weight classification		
Adequate weight	3	2.6
Insufficient weight	13	11.4
Low weight	82	71.9
Very low weight	12	10.5
Extremely low weight	4	3.5
Preterm classification		
Late preterm newborn	61	53.5
Moderately preterm newborn	30	26.3
Very preterm newborn	19	16.7
Extremely preterm newborn	4	3.5
Hospitalization days		
Up to 30 days	84	73.7
More than 30 days	30	26.3
Type of diet at discharge		
Exclusive breastfeeding	58	50.9
Breastfeeding + formula	45	39.5
Formula	11	9.6

The category content analysis divided the mothers' perceptions of factors that interfere with the newborn's breastfeeding into four semantic categories. Also, a fifth category was created to identify mothers who did not respond to at least one of the questions. Table 2 presents the frequency analysis of the dependent categorical variables that, in the mother's opinion, interfere with breastfeeding. The category with the highest frequency of the mothers’ responses about factors that interfere with newborns’ breastfeeding referred to the mother's clinical, physical, and psychoemotional conditions. The second most mentioned category was the newborns’ clinical or physical conditions, followed by the support network and, lastly, the strategies to start or maintain breastfeeding.

**Table 2:** Frequency analysis of dependent categorical variables that interfere with breastfeeding, according to the mothers’ opinions

Response category	Mothers’ opinions of factors that interfere with breastfeeding	
	Absolute frequency	Relative frequency (%)
Newborn’s clinical and physical conditions	43	37.7
Mother’s clinical, physical, and psychoemotional conditions	48	42.1
Support network	37	32.5
Strategies to start or maintain breastfeeding	18	15.8
No response	30	26.3

**Caption:** n = frequency of the mothers’ responses; % = percentage of the mothers’ responses

Table 3 presents the association between the mothers’ responses about what interferes with breastfeeding and the socioeconomic and pregnancy characteristics and the newborns’ clinical status.

**Table 3:** Result of the association between maternal response about what interferes with breastfeeding and other independent variables

Variables	Newborns’ clinical and/or physical conditions		Mothers’ clinical, physical, and psychoemotional conditions		Support network		Strategies to start and/or maintain breastfeeding		No response	
	Frequency of response	p-value	Frequency of response	p-value	Frequency of response	p-value	Frequency of response	p-value	Frequency of response	p-value
Education level										
Up to incomplete high school	19 (16.7%)	0.500	14 (12.3%)	0.001*	17 (14.9%)	0.733	8 (7.0%)	0.725	20 (17.5%)	0.019*
Complete high school or higher education	24 (21.0%)		34 (29.8%)		20 (17.5%)		10 *8.8%)		10 (8.8%)	
Works outside the home										
Yes	17 (14.9%)	0.890	15 (13.2%)	0.091	16 (14.0%)	0.663	10 (8.8%)	0.152	16 (14.0%)	0.411
No	26 (22.8%)		33 (28.9%)		21 (18.4%)		8 (7.0%)		14 (12.3%)	
The father is present										
Yes	37 (32.5%)	0.226	48 (42.1%)	0.003*	36 (31.6%)	0.082	18 (15.8%)	0.131	26 (22.8%)	0.426
No	6 (5.3%)		0		1 (0.9%)		0		4 (3.5%)	
Primiparity										
Primiparous	17 (14.9%)	0.112	20 (17.5%)	0.030*	7 (6.1%)	0.059	5 (4.4%)	0.769	5 (4.4%)	0.052
Multiparous	26 (22.8%)		28 (24.6%)		30 (26.3%)		13 (11.4%)		25 (21.9%)	
Breastfed previous children										
Yes	23 (20.2%)	0.397	23 (20.2%)^A^	0.029*	23 (20.2%)	0.294	10 (8.8%)	0.611	24 (21.0%)	0.051
No	3 (2.6%)		5 (4.4%)^B^		4 (3.5%)		2 (1.7%)		1 (0.9%)	
Primiparous	17 (14.9%)		20 (17.5%)^B^		10 (8.8%)		6 (5.3%)		5 (4.4%)	
Birth weight classification										
Adequate weight	1 (0.9%)	0.403	2 (1.7%)	0.295	2 (1.7%)	0.511	0	0.332	1 (0.9%)	0.993
Insufficient weight	2 (1.7%)		6 (5.3%)		5 (4.4%)		2 (1.7%)		4 (3.5%)	
Low weight	35 (30.7%)		30 (26.3%)		27 (23.7%)		13 (11.4%)		21 (18.4%)	
Very low weight	4 (3.5%)		8 (7.0%)		2 (1.7%)		1 (0.9%)		3 (2.6%)	
Extremely low weight	1 (0.9%)		2 (1.7%)		1 (0.9%)		2 (1.7%)		1 (0.9%)	
Preterm classification										
Late preterm NB	21 (18.4%)	0.711	21 (18.4%)	0.237	23 (20.2%)	0.152	9 (7.9%)	0.841	18 (15.8%)	0.815
Moderately preterm NB	12 (10.5%)		15 (13.2%)		11 (9.65%)		4 (3.5%)		6 (5.3%)	
Very preterm NB	9 (7.9%)		9 (7.9%)		2 (1.7%)		4 (3.5%)		5 (4.4%)	
Extremely preterm NB	1 (0.9%)		3 (2.6%)		1 (0.9%)		1 (0.9%)		1 (0.9%)	
Hospitalization days										
Up to 30 days	29 (25.4%)	0.239	35 (30.7%)	0.874	31 (27.2%)	0.090	14 (12.3%)	0.667	22 (19.3%)	0.959
More than 30 days	14 (12.3%)		13 (11.4%)		6 (5.3%)		4 (3.5%)		8 (7.02%)	
Type of diet at discharge										
Exclusive breastfeeding	22 (19.3%)	0.995	25 (21.9%)^A^	0.917	25 (21.9%)	**0.011** *	11 (9.6%)	0.541	13 (11.4%)	0.281
breastfeeding + formula	17 (14.9%)		19 (16.7%)^AB^		12 (10.5%)		5 (4.4%)		12 (10.5%)	
Formula	4 (3.5%)		4 (3.5%)^B^		0		2 (1.7%)		5 (4.4%)	

Different superscript letters indicate a statistical difference

* Significant p-value < 0.05

**Caption:** NB = newborn. Pearson’s chi-square test

The mothers’ perception that their clinical, physical, and psychoemotional conditions interfere with breastfeeding was associated with higher education levels, paternal presence, and multiparity. Such maternal perception was also associated with the experience of breastfeeding other children. The analysis between categories revealed a difference in this perception between mothers who breastfed previous children and those who did not breastfeed (p = 0.043) and between those who breastfed previous children and primiparous women (p = 0.033).

The mothers' responses regarding the support network as a factor interfering with breastfeeding were associated with the type of diet at discharge. The analysis between the categories revealed a difference in this perception between mothers of babies who used formula and those whose babies were on exclusive breastfeeding (p = 0.006). Most newborns whose mothers pointed out that the support network favors breastfeeding or that the lack of it makes the process difficult were exclusively breastfeeding at discharge.

The lack of response from mothers regarding their perceptions of factors that may interfere with breastfeeding was associated with lower education levels. The other associations investigated were not significant.

Table 4 compares the mothers’ ages, according to whether they pointed out any interfering factor in the different categories. There was no significant difference in their ages in any of the categories.

**Table 4:** Comparison between the mothers’ ages per response category. according to their responses

Response categories	Mothers who pointed out this category	Mothers who did not point out this category	p-value
	Mean age ± standard deviation	Mean age ± standard deviation	
Newborn’s clinical and/or physical conditions	26.5 ±7.8	29.0±7.7	0.094
Mothers’ clinical, physical, and/or psychoemotional conditions	26.8±7.1	29.0±8.2	0.201
Support network	29.7±7.8	27.3±7.8	0.104
Strategies to start and/or maintain breastfeeding	28.4±8.8	28.0±7.7	0.988
No response	29.3±8.5	27.6±7.6	0.422

Kruskal-Wallis test. Significant p-value < 0.05

## DISCUSSION

The mothers’ opinion was that their clinical, physical, and psychoemotional conditions were the most influential factor in preterm newborns’ exclusive breastfeeding. In addition to biological aspects, breastfeeding is known to be part of the newborn's mothering^(16)^, involving the woman's emotions and perceptions of this experience^(17)^. A study investigated how they coped with mothering preterm children and found that it was a difficult, distressing, frustrating experience, with the insecurity of caring for preterm children and their physiological weaknesses^(16)^. Moreover, the newborn’s removal to intensive care generates maternal distress^(16)^. A survey with mothers of preterm newborns admitted to a NICU reported that they considered it a particularly stressful environment, where parents are separated from the infant, with no privacy, and restricted feeding times prone to anxiety – which affects lactogenesis^(18)^. The largest number of responses in the category of mothers' clinical, physical, and psychoemotional conditions is probably due to the research being carried out during hospitalization, which is permeated by such feelings of anguish, anxiety, and tiredness.

Furthermore, the education level was associated with the mothers’ report that their clinical, physical, and psychoemotional conditions influence breastfeeding. The higher the education level, the greater their perception that their clinical, physical, and psychoemotional conditions influence exclusive breastfeeding. This can be explained by these mothers’ higher levels of education and, consequently, greater knowledge about breastfeeding and its importance for them and their newborns. This fact was also highlighted in the study by Amando and collaborators (2016)^(19)^, which aimed to analyze the mothers' perceptions of breastfeeding preterm newborns admitted to a neonatal intermediate and intensive care unit. It revealed that mothers recognize the importance of breastfeeding preterm children but point out difficulties in breastfeeding them while hospitalized^(19)^. The authors reported that greater maternal education helps them assimilate more easily the information they receive about breastfeeding^(19)^. Another study shows that mothers with more than 8 years of education had greater interest in exclusive breastfeeding than those with fewer years of education^(20)^.

Regarding paternal presence, all mothers who considered their own conditions as influencing breastfeeding reported that the newborn's father was present. This finding suggests that the father's participation at this point in the postpartum period allows the woman to reflect on her own clinical, physical, and psychoemotional conditions and the effect of her physical and emotional health status on milk production and let-down, possibly because they received support or had the perception that someone was present to help care for the preterm baby, including feeding. Regarding this topic, studies show that the father's positive attitude has a motivating effect on the mother to breastfeed^(9,21,22)^, especially in the first months of life^(15)^.

Most mothers who considered their clinical, physical, and psychoemotional conditions as influencing the newborn's feeding at discharge were multiparous and breastfed previous children. The literature points out that the lack of previous breastfeeding experiences is a predictive factor for the interruption of exclusive breastfeeding^(23)^, while mothers with positive previous experiences tend to have an easier time establishing breastfeeding with their other children^(9)^. The results of this research suggest that the experience of breastfeeding previous children brings more awareness to mothers about the possibility of their physical, clinical, and psychoemotional conditions influencing the newborn's feeding.

Most mothers who responded that the support network influences breastfeeding were exclusively breastfeeding at discharge. It is known that the agents that make up the maternal support network are important to establish and maintain breastfeeding^(24)^. Health professionals clarify questions and help to reduce fears and anxieties^(19)^. Studies state that their work, clarifications, and provision of information provide parents with relief and hope^(25)^. Studies also address the family support network as an important provider of material support, with domestic care and specific care for preterm newborns and emotional support for mothers during the postpartum period^(26)^. The fact that they mention support networks suggests the current presence or previous experience with an effective support network. It is important to mention that this study was conducted in a baby-friendly hospital, where health professionals provide breastfeeding support and assistance when conditions allow this form of feeding.

Most mothers who did not report their perceptions of factors that interfere with breastfeeding had not completed high school or higher education, suggesting less access to information and less capacity for reflection^(9)^. A study aimed to analyze the level of functional health literacy of users of family health units in the urban area of Altamira, in Pará, Brazil, and identified that low education increases the risk of individuals having unsatisfactory functional health literacy, corroborating the findings of this study^(27)^.

In view of the results analyzed and discussed, the limitations of this study include contacting mothers at a delicate postpartum moment, when they were coping with the emotions of premature birth and their newborns’ other weaknesses. It is suggested that future research redo the interview at another time. Moreover, it was difficult to contact and monitor these families after discharge to further encourage exclusive breastfeeding until the sixth month of life. The strengths of the study include its qualitative and quantitative data analyses and the encouragement of breastfeeding provided during hospitalization.

## CONCLUSION

Education level, paternal presence, having other children, and having breastfed them were associated with mothers' opinions of whether their clinical, physical, and psychoemotional conditions interfere with breastfeeding. Mentioning the importance of the support network was associated with exclusive breastfeeding at discharge. Furthermore, lower education levels were associated with the lack of response to the questionnaire about their perception of factors that influence the feeding of preterm babies.
