# Supplementary material for: Maternal perception of factors that interfere with breastfeeding of preterm newborns
Source: Codas. 2024 Aug 5;36(5):e20230252. doi: 10.1590/2317-1782/20242023252en (PMC11340869; doi:10.1590/2317-1782/20242023252en)
Supplement: Translation

# Translation: Percepção materna sobre os fatores que interferem na amamentação de prematuros

**DOI:** 10.1590/2317-1782/20242023252pt

**Authors:** Bárbara Helem da Fonseca Patrocínio Werneck, Juliana Cordeiro de Oliveira, Cláudia Gonçalves de Oliveira, Andréa Rodrigues Motta, Amélia Augusta de Lima Friche, Renata Maria Moreira Moraes Furlan

**Affiliations:** 1 Graduação em Fonoaudiologia, Universidade Federal de Minas Gerais – UFMG - Belo Horizonte (MG), Brasil.; 2 Programa de Pós-graduação em Ciências Fonoaudiológicas, Universidade Federal de Minas Gerais - UFMG - Belo Horizonte (MG), Brasil.; 3 Departamento de Fonoaudiologia, Universidade Federal de Minas Gerais – UFMG - Belo Horizonte (MG), Brasil.

**Keywords:** Aleitamento Materno, Recém-nascido Prematuro, Neonatologia, Fonoaudiologia, Saúde Materno-infantil

## Abstract

**Objetivo:**

verificar a associação entre a percepção das mães de prematuros a respeito dos fatores que podem interferir no aleitamento e as características socioeconômicas da mãe, da gestação e clínicas do recém-nascido.

**Método:**

estudo qualiquantitativo observacional, descritivo e analítico do tipo transversal. Foram incluídas 114 mães de prematuros. Os dados foram coletados por meio de questionários, aplicados à alta hospitalar e análise dos prontuários. As respostas maternas sobre as interferências observadas no processo do aleitamento foram categorizadas por análise de conteúdo e associadas aos dados socioeconômicos, da gestação e do recém-nascido (RN).

**Resultados:**

as percepções das mães quanto aos fatores que interferem na alimentação do RN ao seio materno foram divididas em quatro categorias semânticas: condições clínicas e/ou físicas do RN; condições clínicas, físicas e/ou psicoemocionais da mãe; rede de apoio; e estratégias para iniciar e/ou manter o aleitamento materno. Escolaridade, presença paterna, possuir outros filhos e tê-los amamentado apresentaram associação com a percepção materna de que suas condições clínicas, físicas e ou psicoemocionais interferem no aleitamento. Além disso, a rede de apoio esteve associada ao aleitamento materno exclusivo à alta.

**Conclusão:**

escolaridade, presença paterna, multiparidade e ter amamentado filhos anteriores influenciaram a percepção materna de que suas condições clínicas, físicas e ou psicoemocionais apresentam interferência no aleitamento. Além disso, a menção à rede de apoio esteve associada com o aleitamento materno exclusivo à alta.

## INTRODUÇÃO

A prematuridade é fator de risco para a morbimortalidade infantil^(1)^. O neonato prematuro é aquele cujo nascimento deu-se antes de se completar 37 semanas de gestação^(2)^. Pode ser classificado, de acordo com a idade gestacional, em prematuro moderado a tardio (32 a 36 semanas e 6 dias de idade gestacional), muito prematuro (28 a 31 semanas e 6 dias de idade gestacional) e extremamente prematuro (idade gestacional inferior a 28 semanas)^(2)^. A idade gestacional muito baixa, bem como a asfixia, o baixo peso ao nascimento e a ocorrência de sepse configuram-se como os fatores de maior risco para um mau prognóstico e até mesmo para a mortalidade infantil^(3)^.

O desenvolvimento que deveria acontecer ainda no útero é interrompido com o nascimento prematuro e, por isso, grande parte destes recém-nascidos (RN) necessitam de cuidados de diversos profissionais para se adaptar ao ambiente externo^(4)^. Em uma unidade neonatal, um dos desafios ao cuidado dos recém-nascidos prematuros tange a alimentação, haja vista que estes recém-nascidos podem apresentar certas limitações nutricionais e fisiológicas no sistema digestivo^(1)^.

O aleitamento materno diminui a morbidade e a mortalidade infantil, garante a intensificação do vínculo entre a mãe e o seu filho^(5)^ e promove o desenvolvimento adequado do sistema estomatognático e de suas funções, tais quais: sucção, respiração, mastigação, deglutição e articulação da fala^(6)^. A alimentação da criança ao seio materno propicia crescimento craniofacial harmônico, bem como equilíbrio da musculatura intra e extraoral^(6)^.

O aleitamento materno pode se iniciar mais tardiamente em RN pré-termo e ter menor duração, quando comparado com o RN a termo^(7)^. O acompanhamento às mães é importante para o sucesso do aleitamento materno, principalmente nos primeiros dias de vida e de internação do RN, mas, também, após a alta hospitalar^(8)^. Existem alguns fatores que podem interferir na prática da amamentação, independente da prematuridade, tais como a idade, situação socioeconômica, condições de trabalho e psicoemocionais da mãe, condições clínicas do RN e as rotinas hospitalares, bem como a rede de apoio materna, envolvendo o pai, familiares, amigos e profissionais de saúde^(9)^.

No que concerne à prematuridade, em dois hospitais amigos da criança da região Sudeste do Brasil, verificou-se que o aleitamento materno exclusivo à alta hospitalar foi influenciado pelo estado civil, ocupação materna, número de consultas de pré-natal, tipo de parto, idade gestacional, peso ao nascer, tempo de internação e uso de ventilação mecânica^(10)^. Em hospital da rede pública de Maceió, a idade materna maior que 35 anos constituiu fator de proteção para o aleitamento materno exclusivo em prematuros, enquanto a via de parto cesariana foi fator de risco^(11)^. Já em uma maternidade do Paraná, a prematuridade extrema e o uso de chupeta foram fatores preditivos de desmame precoce (antes do sexto mês)^(12)^. Bebês com maior grau de prematuridade tiveram menos chances de aleitamento materno exclusivo à alta hospitalar, assim como aqueles com menor peso ao nascimento, maior tempo de internação e que fizeram uso de ventilação mecânica^(13,14)^.

Tendo em vista a existência de fatores que favorecem ou dificultam a prática da amamentação*,* entender as opiniões das mães de RN prematuros sobre quais são esses fatores, torna-se imprescindível, a fim de buscar estratégias para abordá-las diretamente em suas necessidades, aumentando, assim, as chances de estabelecer e manter o aleitamento materno dessas crianças*.*

Dessa forma, este estudo busca verificar a associação entre a percepção das mães a respeito dos fatores que podem interferir no processo de alimentação de prematuros ao seio materno e as características socioeconômicas da mãe, da gestação e clínicas do recém-nascido.

## MÉTODO

Este é um estudo observacional descritivo e analítico, do tipo transversal qualiquantitativo, realizado na Unidade Neonatal de um Hospital Público, localizado em Betim, Minas Gerais. Participaram do estudo 114 mães de recém-nascidos pré-termo, sem delimitação de idade para serem incluídas.

O estudo foi aprovado pelo Comitê de Ética em Pesquisa da Universidade Federal de Minas Gerais sob o número do parecer 3.589.241 e, também, pelo Comitê de Ética em Pesquisa da Prefeitura da cidade de Betim, Minas Gerais, sob o número do parecer 4.222.766. Todas as participantes do estudo assinaram o Termo de Consentimento Livre e Esclarecido ou o Termo de Assentimento.

A coleta de dados foi realizada entre outubro de 2019 a março de 2020, considerando como critérios de inclusão: o filho ter nascido na maternidade do Hospital Público Regional Professor Osvaldo Franco, ser prematuro (nascido com menos que 37 semanas de idade gestacional), ter permanecido no mínimo 48 horas internado em Unidade de Terapia Intensiva Neonatal (UTIN) do referido hospital e a mãe ter o desejo de amamentar. Os critérios de exclusão considerados foram: o filho ter sido transferido para outra instituição; apresentar diagnóstico suspeito ou confirmado de síndromes, hemorragias peri ou intraventricular graus III e IV, ou ainda, apresentar má-formação craniofacial ou dos órgãos do sistema digestivo (por exemplo, fissura labiopalatina ou atresia de esôfago), ou alterações na absorção e digestão de leite materno (por exemplo, galactosemia do lactente); a mãe ter HIV/AIDS ou incapacidade cognitiva para responder ao questionário. Essas informações foram obtidas nos prontuários hospitalares.

Foram coletadas informações sobre as características socioeconômicas das mães, tais quais: idade; escolaridade (sem estudo, ensino fundamental incompleto, ensino fundamental completo, ensino médio incompleto, ensino médio completo, ensino superior incompleto ou ensino superior completo); se exerce ou não função remunerada e se há presença paterna ou não no cotidiano. Também foram coletados dados sobre a gestação (o número de gestações anteriores e se amamentou os outros filhos) e foram obtidos, próximo ao momento da alta hospitalar, o relato das opiniões das mães sobre os fatores que acreditam interferir na alimentação do RN ao seio materno, favorecendo ou dificultando a amamentação. Os dados do RN, quanto ao peso, idade gestacional ao nascer, dias de internação e dieta à alta, foram coletados nos prontuários. A idade gestacional dos RN ao nascimento serviu de parâmetro para a classificação do grau de prematuridade, segundo as subcategorias da Organização Mundial de Saúde^(2)^.

As informações coletadas foram organizadas e armazenadas em uma planilha Excel^®^. Foi utilizada a Técnica de Análise Categorial da Análise de Conteúdo, segundo Bardin^(15)^, para a análise das respostas das mães em relação aos fatores de interferência na alimentação dos RN, por serem questões abertas. A Análise Categorial consiste no desmembramento do texto em unidades menores, que podem ser palavras, frases ou expressões^(15)^.

A análise de conteúdo possui três fases: pré-análise; exploração do material, categorização ou codificação; tratamento dos resultados, inferências e interpretação^(15)^. Foi realizada uma pré-análise dos dados, na qual eles foram transcritos para uma planilha e lidos integralmente. Para a definição das categorias, foram consideradas as palavras ou expressões citadas de maneira repetitiva pelas mães. Após a identificação dos assuntos mais abordados, os resultados foram tratados e definiram-se as quatro categorias semânticas que reúnem as opiniões maternas. Ao final, cada resposta poderia ser classificada em mais de uma categoria, a depender de quais eram os diferentes fatores que as mães consideravam interferir na amamentação ao seio materno.

A análise estatística descritiva das variáveis foi efetuada por meio de tabelas de distribuição de frequências, absoluta e relativa, para as variáveis categóricas, e síntese numérica das variáveis quantitativas. As variáveis dependentes foram as respostas das mães, referentes às suas percepções sobre os fatores que interferem na amamentação. Foram consideradas variáveis independentes: idade materna, escolaridade materna, atividade profissional, presença paterna, primiparidade, experiência prévia com amamentação, classificação de peso, classificação do grau de prematuridade, dias de internação e tipo de dieta à alta.

Inicialmente utilizou-se o teste Shapiro-Wilk para verificação do tipo de distribuição dos dados, sendo verificada ausência de características de normalidade. Utilizou-se o Teste Qui-quadrado de Pearson para verificar a associação entre as variáveis independentes categóricas e as variáveis-resposta, relacionadas às respostas da mãe, e o teste Kruskal-Wallis para averiguar a relação entre a idade da mãe e as respostas. Adotou-se um nível de significância de 5% em todas as análises. As categorias da variável escolaridade foram agrupadas em: até ensino médio incompleto, que englobava o ensino fundamental incompleto e completo e o ensino médio incompleto; e médio completo ou superior, que abarcava o ensino médio completo e o superior incompleto ou completo.

## RESULTADOS

Participaram do estudo 114 mães, cujas idades variaram entre 15 e 44 anos, sendo a média 28,1 anos e o desvio-padrão (DP) de 7,8 anos. A média do número de gestações anteriores foi de 1,6 gestações (DP=1,7), sendo o mínimo 0 e o máximo 9.

Na Tabela 1 é apresentada a análise de frequência das variáveis independentes do estudo. A maior parte das mães possui ensino médio completo, relatou não trabalhar fora de casa, informou que o pai da criança era presente, possuía outros filhos e confirmou ter amamentado os filhos anteriores. Quanto aos recém-nascidos, a maioria era pré-termo tardio, apresentava baixo peso, permaneceu internado até 30 dias e pouco mais da metade recebeu alta em aleitamento materno exclusivo.

**Tabela 1:** Análise descritiva das características das mães e dos RN prematuros

Variável categórica	Frequência absoluta	Frequência relativa (%)
Escolaridade		
Fundamental incompleto	12	10,5
Fundamental completo	10	8,8
Médio incompleto	33	28,9
Médio completo	57	50,0
Superior completo ou incompleto	2	1,7
Trabalho fora de casa		
Sim	46	40,3
Não	68	59,6
Pai presente		
Sim	103	90,3
Não	11	9,6
Primiparidade		
Primíparas	35	30,7
Multíparas	79	69,3
Amamentou filhos anteriores		
Sim	70	61,4
Não	7	6,1
Não se aplica	37	32,5
Classificação do peso ao nascimento		
Peso adequado	3	2,6
Peso insuficiente	13	11,4
Baixo peso	82	71,9
Muito baixo peso	12	10,5
Extremo baixo peso	4	3,5
Classificação do grau de prematuridade		
RNPT tardio	61	53,5
RNPT moderado	30	26,3
RNPT muito prematuro	19	16,7
RNPT extremo	4	3,5
Dias de internação		
Até 30 dias	84	73,7
Acima de 30 dias	30	26,3
Tipo de dieta à alta		
Aleitamento materno exclusivo	58	50,9
Aleitamento materno + fórmula láctea	45	39,5
Fórmula láctea	11	9,6

**Legenda:** RNPT=recém-nascido prematuro

Como resultado da análise categorial da análise de conteúdo, as percepções das mães quanto aos fatores que interferem na alimentação do RN ao seio materno foram divididas em quatro categorias semânticas e foi criada uma quinta categoria para identificar as mães que não responderam a, pelo menos, uma das perguntas. A Tabela 2 apresenta a análise de frequência dessas variáveis categóricas dependentes que, na opinião materna, interferem no aleitamento materno. A categoria com maior frequência de resposta das mães, quanto aos fatores de interferência na amamentação dos RN, foi a que se refere às condições clínicas, físicas ou psicoemocionais da mãe. Logo após, as condições clínicas ou físicas do RN foi a categoria mais referida, seguida pela rede de apoio e, por fim, as estratégias para iniciar ou manter o aleitamento materno.

**Tabela 2:** Análise de frequência das variáveis categóricas dependentes que, na opinião materna, interferem no aleitamento

Categoria de respostas	Opinião das mães quanto aos fatores que interferiram	
	Frequência absoluta	Frequência relativa (%)
Condições clínicas ou físicas do RN	43	37,7
Condições clínicas, físicas ou psicoemocionais da mãe	48	42,1
Rede de apoio	37	32,5
Estratégias para iniciar ou manter o aleitamento materno	18	15,8
Não respondeu	30	26,3

**Legenda:** n= frequência de respostas das mães; % = percentual das respostas das mães. RN = recém-nascido

A Tabela 3 apresenta a associação entre as respostas maternas sobre o que interfere no aleitamento materno e as características socioeconômicas, de gestação e clínicas do recém-nascido.

**Tabela 3:** Resultado da associação entre a resposta materna sobre o que interfere no aleitamento e demais variáveis independentes

Variáveis	Condições clínicas e/ou físicas do RN		Condições clínicas, físicas e psicoemocionais da mãe		Rede de apoio		Estratégias para iniciar e/ou manter o aleitamento materno		Não respondeu	
	Frequência de resposta	Valor de p	Frequência de resposta	Valor de p	Frequência de resposta	Valor de p	Frequência de resposta	Valor de p	Frequência de resposta	Valor de p
Escolaridade										
Até Médio incompleto	19 (16,7%)	0,500	14 (12,3%)	0,001*	17 (14,9%)	0,733	8 (7,0%)	0,725	20 (17,5%)	0,019*
Médio completo ou superior	24 (21,0%)		34 (29,8%)		20 (17,5%)		10 *8,8%)		10 (8,8%)	
Trabalho fora de casa										
Sim	17 (14,9%)	0,890	15 (13,2%)	0,091	16 (14,0%)	0,663	10 (8,8%)	0,152	16 (14,0%)	0,411
Não	26 (22,8%)		33 (28,9%)		21 (18,4%)		8 (7,0%)		14 (12,3%)	
Pai presente										
Sim	37 (32,5%)	0,226	48 (42,1%)	0,003*	36 (31,6%)	0,082	18 (15,8%)	0,131	26 (22,8%)	0,426
Não	6 (5,3%)		0		1 (0,9%)		0		4 (3,5%)	
Primiparidade										
Primíparas	17 (14,9%)	0,112	20 (17,5%)	0,030*	7 (6,1%)	0,059	5 (4,4%)	0,769	5 (4,4%)	0,052
Multíparas	26 (22,8%)		28 (24,6%)		30 (26,3%)		13 (11,4%)		25 (21,9%)	
Amamentou filhos anteriores										
Sim	23 (20,2%)	0,397	23 (20,2%)^A^	0,029*	23 (20,2%)	0,294	10 (8,8%)	0,611	24 (21,0%)	0,051
Não	3 (2,6%)		5 (4,4%)^B^		4 (3,5%)		2 (1,7%)		1 (0,9%)	
Primíparas	17 (14,9%)		20 (17,5%)^B^		10 (8,8%)		6 (5,3%)		5 (4,4%)	
Classificação do peso ao nascimento										
Peso adequado	1 (0,9%)	0,403	2 (1,7%)	0,295	2 (1,7%)	0,511	0	0,332	1 (0,9%)	0,993
Peso insuficiente	2 (1,7%)		6 (5,3%)		5 (4,4%)		2 (1,7%)		4 (3,5%)	
Baixo peso	35 (30,7%)		30 (26,3%)		27 (23,7%)		13 (11,4%)		21 (18,4%)	
Muito baixo peso	4 (3,5%)		8 (7,0%)		2 (1,7%)		1 (0,9%)		3 (2,6%)	
Extremo baixo peso	1 (0,9%)		2 (1,7%)		1 (0,9%)		2 (1,7%)		1 (0,9%)	
Classificação do grau de prematuridade										
RNPT tardio	21 (18,4%)	0,711	21 (18,4%)	0,237	23 (20,2%)	0,152	9 (7,9%)	0,841	18 (15,8%)	0,815
RNPT moderado	12 (10,5%)		15 (13,2%)		11 (9,65%)		4 (3,5%)		6 (5,3%)	
RNPT muito prematuro	9 (7,9%)		9 (7,9%)		2 (1,7%)		4 (3,5%)		5 (4,4%)	
RNPT extremo	1 (0,9%)		3 (2,6%)		1 (0,9%)		1 (0,9%)		1 (0,9%)	
Dias de internação										
Até 30 dias	29 (25,4%)	0,239	35 (30,7%)	0,874	31 (27,2%)	0,090	14 (12,3%)	0,667	22 (19,3%)	0,959
Acima de 30 dias	14 (12,3%)		13 (11,4%)		6 (5,3%)		4 (3,5%)		8 (7,02%)	
Tipo de dieta à alta										
LME	22 (19,3%)	0,995	25 (21,9%)^A^	0,917	25 (21,9%)	**0,011** *	11 (9,6%)	0,541	13 (11,4%)	0,281
LM + fórmula láctea	17 (14,9%)		19 (16,7%)^AB^		12 (10,5%)		5 (4,4%)		12 (10,5%)	
Fórmula láctea	4 (3,5%)		4 (3,5%)^B^		0		2 (1,7%)		5 (4,4%)	

Letras sobrescritas diferentes indicam diferença estatística

* Valor de p significativo <0,05

**Legenda:** RNPT = recém-nascido prematuro; LME = leite materno exclusivo; LM + fórmula láctea = leite materno associado à fórmula láctea. Teste Qui-quadrado de Pearson.

Houve associação entre a percepção materna de que as condições clínicas, físicas ou psicoemocionais da mãe interferem no aleitamento materno e a maior escolaridade, a presença paterna e a multiparidade. Também houve associação dessa percepção materna com a experiência de amamentar outros filhos. A análise entre as categorias revelou diferença nesta percepção entre mães que amamentaram filhos anteriores e que não amamentaram (p=0,043) e entre as que amamentaram filhos anteriores e as primíparas (p=0,033).

As respostas das mães quanto à rede de apoio como fator de interferência na alimentação ao seio materno apresentaram associação com o tipo de dieta à alta. A análise entre as categorias revelou diferença nesta percepção entre as mães de bebês que usavam fórmula e aquelas cujo bebê se encontrava em aleitamento materno exclusivo (p=0,006), sendo que a maioria dos RN, cujas mães apontaram que a rede de apoio favorece a amamentação ou que a falta dela dificulta o processo, estava em aleitamento materno exclusivo à alta.

A ausência de resposta das mães quanto às suas percepções sobre os fatores que podem interferir no aleitamento materno esteve associada à menor escolaridade. As demais associações investigadas não apresentaram significância.

A Tabela 4 apresenta a comparação das idades maternas, conforme ter apontado ou não algum fator de interferência da respectiva categoria em sua resposta. Não houve diferença significativa de idade materna para nenhuma das categorias.

**Tabela 4:** Comparação entre as idades maternas, conforme as respostas das mães, para cada categoria de resposta

Categorias de respostas	Mães que apontaram essa categoria	Mães que não apontaram essa categoria	Valor de p
	Média de idade ± desvio padrão	Média de idade ± desvio padrão	
Condições clínicas e/ou físicas do RN	26,5 ±7,8	29,0±7,7	0,094
Condições clínicas, físicas e/ou psicoemocionais da mãe	26,8±7,1	29,0±8,2	0,201
Rede de apoio	29,7±7,8	27,3±7,8	0,104
Estratégias para iniciar e/ou manter o aleitamento materno	28,4±8,8	28,0±7,7	0,988
Não respondeu	29,3±8,5	27,6±7,6	0,422

Teste Kruskal-Wallis. Valor de p significativo <0,05

## DISCUSSÃO

As condições clínicas, físicas ou psicoemocionais das mães foram consideradas como fator de maior influência no aleitamento materno exclusivo de RN prematuros, segundo a opinião das próprias mães. Sabe-se que, além dos aspectos biológicos, a amamentação faz parte da maternagem do RN^(16)^ e envolve as emoções da mulher e a forma com que ela encara essa experiência^(17)^. Um estudo investigou o enfrentamento de maternar filho prematuro e verificou que as mães consideram a experiência difícil, angustiante, frustrante e passam pela insegurança de cuidar de um prematuro e suas fragilidades fisiológicas^(16)^. Além disso, o afastamento do recém-nascido para cuidados intensivos também gera angústia materna^(16)^. Uma pesquisa realizada com mães de RN prematuros internados em uma UTI Neonatal registrou que as mães consideram a UTI Neonatal um ambiente particularmente estressante, onde os pais encontram-se separados do lactente, sem privacidade, com horários restritos de alimentação e propício à ansiedade, condição que afeta a lactogênese^(18)^. Provavelmente, o maior número de respostas na categoria das condições clínicas, físicas ou psicoemocionais das mães ocorreu por ter sido a pesquisa realizada no período de internação, momento permeado por tais sentimentos de angústia, ansiedade e cansaço.

Com relação aos fatores associados às respostas maternas, a escolaridade esteve associada ao relato de que condições clínicas, físicas e psicoemocionais da mãe influenciam a amamentação. Quanto maior a escolaridade, maior foi a percepção materna quanto à influência das condições clínicas, físicas ou psicoemocionais das mães no aleitamento materno exclusivo, o que pode ser explicado pelo maior grau de instrução dessas mães e, consequentemente, pelo maior conhecimento sobre o processo do aleitamento materno e a sua importância para a díade. Esse fato também foi pontuado por Amando e colaboradores (2016)^(19)^ em seu estudo, que objetivou analisar a percepção das mães quanto ao processo de amamentação de recém-nascidos pré-termos internados na Unidade Neonatal de Cuidados Intermediários e Intensivos. Tal estudo revelou que as mães reconhecem a importância da amamentação para os filhos prematuros, mas apontam dificuldades para amamentá-los sob hospitalização^(19)^. Os autores apontam que a maior escolaridade materna auxilia a assimilarem com maior facilidade as informações que lhes são passadas sobre a amamentação^(19)^. Nesse contexto, outro estudo aponta que mães com escolaridade superior a oito anos revelaram maior interesse em realizar o aleitamento materno exclusivo em comparação às mães com escolaridade inferior a esse período^(20)^.

Com relação à presença paterna, todas as mães que consideraram suas próprias condições como influenciadoras do aleitamento materno, relataram que o pai do RN era presente. Esse achado sugere que a participação do pai nesse momento do puerpério permite à mulher refletir sobre suas próprias condições (clínicas, físicas e psicoemocionais) e sobre o efeito de seu estado de saúde físico e emocional na produção e descida do leite, possivelmente pelo apoio de fato recebido ou pela ideia de que haverá alguém para auxiliá-la nos cuidados com o prematuro, incluindo a alimentação. Com respeito a essa temática, estudos abordam que a atitude positiva do pai exerce efeito de motivação para a mãe amamentar^(9,21,22)^, especialmente nos primeiros meses de vida^(15)^.

A maior parte das mães que consideraram suas condições clínicas, físicas ou psicoemocionais como influenciadoras da alimentação do RN à alta era multípara e amamentou filhos anteriores. A literatura aponta que a falta de experiência prévia com a amamentação é um fator preditivo para a interrupção do aleitamento materno exclusivo^(23)^, enquanto mães com experiência prévia positiva tendem a ter mais facilidade para estabelecer o aleitamento materno com os demais filhos^(9)^. Os resultados da presente pesquisa sugerem que a experiência de amamentar filhos anteriores traz mais consciência às mães sobre a possibilidade de suas condições físicas, clínicas ou psicoemocionais influenciarem a alimentação do RN.

A maioria das mães que responderam que a rede de apoio influencia na amamentação estava em aleitamento materno exclusivo à alta. Sabe-se que os agentes que compõem a rede de apoio materna são importantes para o estabelecimento e a manutenção do aleitamento materno^(24)^. Os profissionais de saúde auxiliam na redução de medos e ansiedades, no esclarecimento de dúvidas^(19)^ e, estudos afirmam que a atuação dos profissionais de saúde, seus esclarecimentos e fornecimento de informações, geram alívio e esperança aos pais^(25)^. Para mais, estudos abordam a rede de apoio familiar como fornecedora importante de apoio material, com cuidados domésticos e cuidados específicos com o recém-nascido prematuro, além de apoio afetivo às mães durante o puerpério^(26)^. O fato de mencionarem a rede de apoio sugere a presença atual ou experiência prévia com uma rede de apoio efetiva. É importante mencionar que o estudo foi conduzido em um hospital Amigo da Criança, em que o apoio e o auxílio à amamentação são fornecidos pelos profissionais de saúde, quando as condições permitem essa forma de alimentação.

Sobre as mães que não relataram suas percepções sobre os fatores que interferem no aleitamento materno, a maior parte não apresentava ensino médio completo ou ensino superior, sugerindo um menor acesso às informações e menor capacidade de reflexão^(9)^. Um estudo que visava analisar o nível de letramento funcional em saúde dos usuários de Unidades de Saúde da Família na área urbana da cidade de Altamira, no estado do Paraná, identificou que a baixa escolaridade aumenta o risco de os indivíduos possuírem um letramento funcional em saúde insatisfatório, corroborando os achados deste estudo^(27)^.

Por efeito dos resultados analisados e discutidos, percebe-se como limitações deste estudo o fato de as mães terem sido contatadas em um momento delicado do puerpério, em que precisavam lidar com as emoções de um parto prematuro e as demais fragilidades de seus RN, sendo possível sugerir que pesquisas futuras refaçam a entrevista em outro momento, além da dificuldade de contato e acompanhamento dessas famílias após a alta para fortalecimento do incentivo ao aleitamento materno exclusivo até o sexto mês de vida. Como pontos fortes do estudo, têm-se o fato de a análise dos dados ter sido realizada tanto de forma qualitativa quanto quantitativa e o incentivo ao aleitamento materno que foi fornecido durante a internação.

## CONCLUSÃO

A escolaridade, a presença paterna, possuir outros filhos e tê-los amamentado apresentaram associação com as opiniões das mães quanto às suas condições clínicas, físicas ou psicoemocionais interferirem no aleitamento materno. Mencionar a importância da rede de apoio esteve associada ao aleitamento materno exclusivo à alta. Além disso, o menor grau de escolaridade esteve associado à ausência de resposta ao questionário sobre a própria percepção dos fatores que influenciam na alimentação dos prematuros.
